# Proximate Composition, Amino Acid Profile, and Oxidative Stability of Slow-Growing Indigenous Chickens Compared with Commercial Broiler Chickens

**DOI:** 10.3390/foods9050546

**Published:** 2020-05-01

**Authors:** Antonella Dalle Zotte, Elizabeth Gleeson, Daniel Franco, Marco Cullere, José Manuel Lorenzo

**Affiliations:** 1 Department of Animal Medicine, Production and Health, University of Padova, Agripolis 16, Viale dell’Università, 35020 Legnaro, PD, Italy; elizabethyvonne.gleeson@studenti.unipd.it (E.G.); marco.cullere@unipd.it (M.C.); 2 Centro Tecnológico de la Carne de Galicia, Rúa Galicia No 4, Parque Tecnológico de Galicia, San Cibrán das Viñas, 32900 Ourense, Spain; danielfranco@ceteca.net (D.F.); jmlorenzo@ceteca.net (J.M.L.)

**Keywords:** hybrid, meat quality, lipid oxidation, Polverara breed, poultry, protein oxidation

## Abstract

The increased demand for chicken meat products has led to chickens with increased growth rates and heavier slaughter weights. This has had unintentional negative effects on the genetics of these animals, such as spontaneous, idiopathic muscle abnormalities. There has also been a shift in customer preference towards products from alternative farming systems such as organic and free-range. Indigenous purebred chickens, such as the Polverara, show potential in these systems as they are adapted to more extensive systems. The aim of the present study was to characterize the meat quality traits of the Polverara, by comparing the proximate composition and amino acid profile with that of a commercial Hybrid. In addition, the lipid and protein oxidation was analyzed after eight days of storage. A total of 120 leg meat samples, 60 Polverara and 60 Hybrid were analyzed. Polverara exhibited higher protein content, lower lipid content, and a better amino acid profile. These results indicate that the Polverara has better nutritional meat quality. However, Polverara also showed higher levels of lipid and protein oxidation. Therefore, further research is needed, especially in regards to the fatty acid profile and mineral content of the meat, which is known to affect oxidative stability.

## 1. Introduction

In recent decades there has been an increase in chicken meat consumption. There are numerous reasons for this increase in consumer interest. It is perceived as a healthy source of animal protein, it has lower costs associated with it compared to other meat species, it is suitable for further processing, and there are no religious or cultural constraints associated with its consumption [1]. All these characteristics have contributed to the continuous increase in its consumption. Due to this increased demand, the poultry industry has had to adapt its production strategies. Therefore, the poultry industry has incorporated a set of selection criteria for broiler chickens to meet the demand of the ever-increasing world population [2]. Furthermore, to decrease variability in characteristics such as growth rates and slaughter weights, the chickens used in the poultry industry are obtained from a small number of selected genetic lines. As a consequence, the hybrids used in commercial poultry farming have faster growth rates and increased body weights at slaughter than indigenous purebreds that have not undergone the same genetic selection [3,4,5]. This strategy has mainly focused on production traits, and as a consequence, the intensive selection has had unintentional negative effects on some muscle growth and meat quality attributes of commercial hybrids. Effects such as spontaneous, idiopathic muscle abnormalities and increased susceptibility to stress-induced myopathy are found with increased frequency in fast growing broiler chickens which are typically used in the poultry industry [6,7]. To try and counteract these negative effects, the scientific community has begun to focus on identifying the causes of these abnormalities, and possible breeding strategies that can mitigate these negative effects [8,9]. The research done on indigenous purebred chicken breeds has focused mostly on their role in the conservation of poultry genetics and biodiversity. As indigenous purebred chicken breeds are not commercially used, they do not receive much attention in regards to conservation. The preservation of indigenous purebred populations is mostly limited to hobby or vanity poultry farmers, which has resulted in a progressive loss of biodiversity [10,11]. One possible conservation strategy has been to identify the niche poultry product markets. There is a growing awareness of the health and nutrition regarding animal products amongst consumers. Consequently, there has been an increase in consumer interest in products produced in alternative faming systems such are free range and organic systems [12]. Consumers are willing to pay higher prices for poultry products that are perceived as natural or environmentally friendly, and that are produced on farms with high animal welfare standards and animal nutrition [13]. Indigenous purebred chickens show promise in these types of poultry production systems due to their slower growth rate compared to commercial hybrids and their natural preference for more extensive farming systems. This can be attributed to the fact that they have not been selected for intensive farming and their nature is to be more physically active [7]. One of these indigenous purebred chicken breeds is the Polverara. The Polverara chicken is a medium sized, slow-growing indigenous chicken breed (average slaughter age: 180 days) that has its origin in the Veneto region of Italy [7,14]. Only a small number of studies have been conducted on this breed and its potential for use in the poultry industry and conservation. Recent research such as the studies done by [7,14,15], have been focused on characterizing the breed’s meat quality traits, with the aim of promoting its use in conservation and poultry production. Amino acids are the key constituents of protein, and play important roles as regulators in metabolic pathways which are important for maintenance, growth, reproduction and immunity [16,17]. Protein quality and thus the amino acid profile of the protein contributes to the quality of the meat. Due to the importance of amino acids in optimal animal production and human nutrition, it is beneficial to the scientific advancement to analyze the amino acid profiles of the meat species that are being studied. Another factor that contributes to meat quality is the oxidation of the proteins and lipids. Oxidation causes quality deterioration during meat processing and storage [18]. The alterations due to oxidation can influence the physical and chemical properties of meat including water-holding capacity and meat tenderness. It can also decrease the bioavailability of amino acid residues and the digestibility of the protein. This in turn negatively affects the nutritional value of the meat [19]. The aim of the present study was to assist in characterizing the meat quality traits of the Polverara breed, by comparing the proximate composition and amino acid profile with that of a commercially used Hybrid. In addition, the lipid and protein oxidation was assessed.

## 2. Materials and Methods 

### 2.1. Sampling Procedure and Experimental Groups

The experiment was conducted at the Department of Animal Medicine, Production and Health (MAPS), University of Padova (Italy). A total of *n* = 60 chickens were sampled: *n* = 30 broiler chickens (Hybrid) obtained from a commercial poultry farm, and n = 30 slow-growing, medium sized indigenous chickens (Polverara) which were obtained from the Agricultural Professional High School “Duca degli Abruzzi” (Padova). All chickens were males at their respective slaughter ages, 40 days for the Hybrid group and 180 days for the Polverara group. The samples for both groups were collected on the same day. The farming specifications were reported in the study by [14] and the nutritional composition of the finisher diets fed to the two chicken genotypes is given in Table 1. Hybrids were fed a conventional broiler diet whereas Polverara birds were fed an organic diet for growing chickens. All chickens were processed by an authorized commercial abattoir which consisted of electrical stunning (120 V, 200 Hz) and exsanguination. Thereafter, carcasses underwent soft-scalding (2 min at 53 °C) and evisceration. Subsequently, the carcasses were air-chilled (precooling at 5 °C for 6 min, followed by chilling at 0 °C for 90 min). The legs were then dissected from the carcasses, sorted and collected by a working team. Once sampled, the legs were packaged in food-grade plastic bags and transported in refrigerated conditions (4 ± 1 °C) to the MAPS Department where they were frozen (−18 °C) and transported frozen within 48 h to the Centro Tecnolóxico da Carne (Ourense, Spain) for meat quality evaluations, where the leg samples were thawed during 24 h at 4 ± 1 °C and skinned before analysis. The proximate composition, amino acid profile and day 0 oxidative stability analyses were performed on the right leg samples whereas day 8 oxidative stability analysis was performed on the counterpart samples (left legs).

### 2.2. Proximate Composition

The proximate composition of the right leg samples was evaluated according to International Organization for Standards (ISO), where protein [20], moisture [21] and ash [22] content were determined, while total fat was determined according to the Approved Procedure Am 5–04, established by the American Oil Chemists’ Society [23].

### 2.3. Meat Amino Acid Profile

The amino acid profile of the right leg samples was assessed according to the method described by [24]. In short, a sample (100 mg) in a glass ampoule and 6 N hydrochloric acid solution (5 mL) was mixed, sealed and stored at 110 °C for 24 h. After protein hydrolysis was completed, the hydrolysate was diluted with distilled water (200 mL) and filtered through a 0.45 µm filter (Filter Lab, Barcelona, Spain). Tryptophan content was not determined as it transforms into ammonium under acidic conditions. The derivatization of standards and samples was carried out according to Gálvez et al. [25]. The identification of amino acids was done through high performance liquid chromatography (Alliance 2695 model, Waters, Milford, MA, USA), using a scanning fluorescence detector (model 2475, Waters) according to Munekata et al. [26]. The quantification was done using the external standard technique with amino acid standard (Amino Acid Standard H, Thermo, Rockford, IL, USA). The results are expressed as g per 100 g protein.

### 2.4. Storage Conditions

For oxidative stability analyses, chicken legs (left and right) were individually placed in 300 mm thick polyethylene-ethylene vinyl alcohol-polyethylene (PET-EVOH-PE) trays and were packaged directly by sealing with multilayer PE-EVOH-PE film (74 mm thick, permeability < 2 mL/m^2^ bar/day (Viduca, Alicante, Spain) upon the tray (OVERWRAP) using a heat sealer (LARI3/Pn T-VG-R-SKIN, Ca.Ve.Co., Palazzolo, Italy). The trays were stored at 2 ± 1 °C under light to simulate supermarket conditions, being placed on metal shelving and receiving lux values in the range of 15–20, depending on the tray position (HT 306, Digital luxometer, Italy). The light source was conventional, so any wavelength or range, in this case UV, was not filtered. The samples in the chamber were rotated every 24 h to minimize light intensity differences and possible temperature variations on the surface of the meat. Sixty samples (thirty from each experimental group) were removed from the chamber at 0 (left legs) and 8 (right legs) days of storage for lipid and protein oxidation analysis.

### 2.5. Meat Lipid Oxidation

The lipid and protein oxidation was assessed after 0 and 8 days of storage at 4 °C. Lipid oxidation was assessed using the Thiobarbituric Acid Reactive Substances (TBARS) with the method proposed by [27]. In short, a chicken leg meat sample (2 g) was dispersed in 5% trichloroacetic acid (10 mL) and homogenized with an Ultra-Turrax (IkaT25 basic, Staufen, Germany) for 2 min. The homogenate was kept at −10 °C for 19 min and then centrifuged (2360 g for 10 min). The supernatant was then filtered through a Whatman No. 1 filter paper. The filtrate (5 mL) was mixed with a 0.02 M TBA solution (5 mL) and placed in a water bath (96 °C for 40 min). Thereafter the absorbance was measured at 532 nm. The TBARS value was calculated from a standard curve of malonaldehyde with 1,1,3,3-tetraethoxypropane (TEP) and expressed as mg malonaldehyde per kg of sample.

### 2.6. Meat Protein Oxidation

Protein oxidation was measured with the method outlined by [28] with modifications [29]. Measurements were taken for carbonyl and protein quantification to calculate protein oxidation. A sample (2.5 g) was homogenized with 0.6 M NaCl solution (20 mL) and treated with 10% trichloroacetic acid (1 mL) to obtain a homogenate (100 µL). Thereafter, it was centrifuged for 5 min at 5000 g. The supernatant was derivatized for carbonyl quantification with 2 M HCl (1 mL) with 0.2% 2,4-dinitrophenyl hydrazine (DNPH). For protein quantification 2 M HCl (1 mL) was added. A pellet was obtained and washed with 1:1 ethanol/ethyl acetate (1 mL) three times. It was then dissolved in 20 mM sodium phosphate buffer (1.5 mL) with 6 M guanidine hydrochloride. The carbonyls and protein concentrations were measured with a spectrophotometer at 370 nm and 280 nm, respectively. The protein concentrations were calculated according to a standard curve and bovine serum albumin was used to calculate a protein concentration standard. The results are expressed as nmol carbonyl per mg protein.

### 2.7. Statistical Analysis

All data were analyzed using SAS 9.1.3 statistical software package for Windows (SAS, 2008). Proximate composition and amino acid profile of chicken leg meat were analyzed by a one-way ANOVA testing the effect of the genotype (Hybrid, Polverara). Lipid and protein oxidation of chicken leg meat were analyzed by a two-way ANOVA testing the effects of the genotype and the day of storage (day 0, day 8) as fixed effects, and their interaction. Least square means were obtained using a Bonferroni test, and the significance was calculated at a 5% confidence level.

## 3. Results

### 3.1. Proximate Composition

Results regarding the proximate composition analysis showed that the Polverara leg meat had higher water (*p* = 0.0202), protein (*p* < 0.0001) and ash contents (*p* < 0.0001), and lower lipid content (*p* < 0.0001) when compared to the Hybrid leg meat (Table 2). 

### 3.2. Amino Acid Profile

In regards to the amino acid profile analysis (Table 3), the Polverara leg meat exhibited the highest content for all of the amino acids and significantly higher values were exhibited for some of the essential and non-essential amino acids, which included isoleucine, leucine, phenylalanine, threonine and valine (essential) and glycine, proline and tyrosine (non-essential).

### 3.3. Lipid and Protein Oxidation

Table 4 represents the results of the oxidative status of chicken leg meat evaluated over an 8-day period of refrigerated storage. For lipid oxidation, meat from the Polverara breed exhibited higher levels of oxidation at both day 0 (*p* < 0.0001) and day 8 (*p* < 0.0001) of refrigerated storage compared to meat from the Hybrid chicken. Storage time was also shown to have an effect on TBARS values as its level measured at Day 8 of storage was significantly higher (*p* < 0.0001) than that recorded at day 0. Lipid oxidation exhibited a significant interaction between Genotype and storage time (*p* = 0.0330), as Polverara showed a significant increase in TBARS value from Day 0 to Day 8 and Hybrid did not. Genotype effect was also observed for protein oxidation, where Polverara exhibited higher values at both day 0 (*p* < 0.0001) and day 8 (*p* < 0.0001) of refrigerated storage. In contrast from what was observed for lipid oxidation, protein oxidation was not affected by storage time, observing similar values at day 0 and day 8 of storage. 

## 4. Discussion

Lower lipid and higher protein content was found for Polverara compared to Hybrid. This is possibly attributable to the higher level of locomotory activity, which is characteristic of this breed [7]. Locomotory activity is known to favor myogenesis over lipogenesis [12]. The significantly higher ash content found for Polverara leg meat resides in differences in mineral composition. In particular, it was recently observed that the meat from Polverara legs are unexpectedly rich in heme iron, approximately four times higher than that of hybrid chickens [30]. The Polverara leg meat proximate composition reported in the present study differs to the results reported by [15]. This is attributed to the fact that the latter study included male and female chickens, characterized by marked sexual dimorphism at slaughter age, thus resulting in differences in the average values. Moreover, the Polverara breed has not been subjected to genetic selection for productive performance and meat quality traits and thus more variability is expected when compared to commercially used hybrids. There are variations between the proximate compositions reported for different indigenous chicken breeds [31,32,33,34]. These variations are however expected as the indigenous breeds originated in different geographic locations and have different genetic potential, diets and feeding behaviors. These factors can possibly affect protein and lipid deposition in the meat and can increase the variation between results from studies on different indigenous chicken breeds. This also makes it difficult to compare the results from the present study to studies done on other indigenous breeds.

To the authors knowledge, the study done by [7] is the only previous study which has analyzed the amino acid profile of Polverara breast meat and the present study is the first to analyze the amino acid profile of Polverara leg meat. Both studies confirm the higher content for all the amino acids compared to the Hybrid chicken meat. Factors such as age, affect protein digestibility and deposition, and diet is known to have an effect on the amino acid profile of meat [35]. There are variations among the amino acid analysis results from previous studies involving different indigenous chicken breeds, with some of the studies reporting no differences in amino acid profiles when comparing indigenous chicken breeds to hybrids [32,36]. However, when considering the quantitative contribution of the single amino acid intake per 100 g of meat, the higher content of amino acids in Polverara chicken meat depends on its leanness, irrespective of the age of the animal.

Humans have nine essential amino acids that need to be obtained through their diets [37]. The results indicate that Polverara was the better protein source, as it contained higher amounts of all the amino acids essential in human nutrition, and overall has superior nutritional meat quality compared to the Hybrid. In the case of a human with a body weight of 60 kg, 100 g of Polverara leg meat (vs. 100 g of Hybrid leg meat) contains more of the daily requirements for essential amino acids, with 106% (vs. 102%) for histidine, 84% (vs. 78%) for isoleucine, 70% (vs. 65%) for leucine, 105% (vs. 100%) for lysine, 67% (vs. 62%) for methionine + cysteine, 104% (vs. 94%) for phenylalanine + tyrosine, 99% (vs. 92%) for threonine, and 63% (vs. 59%) for valine.

Oxidation causes quality deterioration in meat and can lead to decreased shelf life of meat products [38]. It also adversely affects the flavor of the meat due to oxidative rancidity which causes “off-flavor” [18]. It is understood that the lipid content and the fatty acid profile of the lipids in meat can affect the oxidative susceptibility of meat [39]. Higher meat lipid content increases oxidative susceptibility. The results from the present study showed that the Polverara leg meat had a lower lipid content compared to the Hybrid. This would suggest that the Polverara leg meat would possibly be less susceptible to oxidation due to the lower lipid content compared to the Hybrid [40]. The findings in the present study depict a different scenario with the Polverara meat exhibiting more oxidation than the Hybrid meat. The observed interaction between Genotype and storage time for TBARS values indicates that the rate of oxidation in Polverara meat is higher than in Hybrid meat. The fatty acid profile, specifically the proportion of polyunsaturated (PUFA) and monounsaturated fatty acids (MUFA), affects the extent of lipid oxidation [18]. Polyunsaturated fatty acids are more susceptible to lipid oxidation than monounsaturated fatty acids, and therefore the extent of lipid oxidation increases with increased proportion of PUFA. Research has shown that this proportion differs between commercial broilers and indigenous chicken breeds [41]. Moreover, these results could also depend on the higher heme iron content found in Polverara meat in previous studies [30]. Heme iron is known to be a catalyst for lipid oxidation [42]. There was significantly more lipid oxidation detected at Day 8 compared to Day 0 for Polverara. This is expected as lipid oxidation increases over time [43]. 

Protein oxidation may cause discoloration of fresh meat and influences quality during storage and processing [19]. The extent and rate of protein oxidation is, among other things, influenced by the amino acid profile, lipid content and quality of the meat. Among the amino acids, cysteine and methionine have the highest oxidative susceptibility [19]. Other amino acids such as tyrosine, phenylalanine, tryptophan, histidine, proline, arginine and lysine are also seen as particularly susceptible to oxidation. The results indicate that the Polverara meat had significantly higher levels of protein oxidation at both Day 0 and Day 8. These results may be explained, in part, by the amino acid profile, in which Polverara exhibited higher concentrations of all the previously mentioned amino acids. The significantly higher level of lipid oxidation found in Polverara may also have contributed to the significantly higher level of protein oxidation.

## 5. Conclusions

Polverara chicken outperformed the commercially used Hybrid in both the proximate composition, where it exhibited higher protein content and lower lipid content, and the amino acid profile, where it exhibited higher content for all of the single amino acids that were analyzed. It did, however, exhibit more lipid and protein oxidation, which could negatively affect the oxidative stability and processing of the meat products. This highlights the need for further research into the meat quality characteristics of the breed, especially in regards to the fatty acid profile and mineral content of the Polverara leg meat. Based on the results obtained until now, the Polverara chicken shows potential as a possible breed to be used in alternative farming systems and in conservation efforts.

## Figures and Tables

**Table 1:** Nutrient composition (g/kg as fed) and energy content (MJ/kg as fed) of finisher diets fed to Hybrid and Polverara chickens.

	Diets	
Nutrient Composition	Hybrid	Polverara
Dry matter (DM)	895	895
Crude protein (CP)	197	168
Ether extract (EE)	71.5	40.7
Crude fiber (CF)	36.8	39.4
Nitrogen-free extract (NFE) ^1^	546	579
Ash	44.5	68.2
Gross energy ^2^	17.6	16.3
Calcium	6.30	14.4
Phosphor	5.65	7.33
L-Lysine	11.9	8.63
DL-Methionine	4.10	4.17

^1^ 100 − (water + crude protein + crude fat + crude fiber + ash). ^2^ (NFE × 4.11) + (CP × 5.64) + (EE × 9.44) + (CF × 4.78) × 10.

**Table 2:** Effect of chicken genotype (Hybrid vs. Polverara) on the proximate composition of leg meat.

	Genotype		*p*-Value	RSD ^1^
	Hybrid	Polverara		
N.	30	30		
Water (%)	72.6	73.7	0.0202	1.67
Protein (%)	18.5	21.5	<0.0001	0.93
Lipids (%)	7.28	2.25	<0.0001	1.69
Ash (%)	1.19	1.31	<0.0001	0.06

^1^ Residual standard deviation.

**Table 3:** Effect of chicken genotype (Hybrid vs. Polverara) on the amino acid profile of leg meat.

	Genotype		*p*-Value	RSD ^1^
	Hybrid	Polverara		
N.	30	30		
*Essential amino acids* *(g/100 g meat)*				
Arginine	1.45	1.55	0.0614	0.21
Histidine	0.61	0.63	0.2686	0.07
Isoleucine	0.94	1.00	0.0273	0.11
Leucine	1.52	1.64	0.0134	0.19
Lysine	1.79	1.90	0.0910	0.24
Methionine	0.35	0.39	0.0719	0.09
Phenylalanine	0.76	0.84	0.0023	0.09
Threonine	0.83	0.89	0.0122	0.09
Valine	0.92	0.98	0.0313	0.10
*Non-essential amino acids* *(g/100 g meat)*				
Alanine	1.09	1.15	0.0533	0.12
Aspartic acid	1.75	1.85	0.1122	0.23
Cysteine	0.21	0.21	0.7863	0.04
Glutamic acid	2.91	3.08	0.0991	0.37
Glycine	0.87	0.95	0.0341	0.15
Proline	0.75	0.84	0.0007	0.10
Serine	0.94	0.95	0.8677	0.28
Tyrosine	0.65	0.71	0.0049	0.08

^1^ Residual standard deviation.

**Table 4:** Effect of genotype (Hybrid vs. Polverara) and day of storage (0 vs. 8) and their interaction on the TBARS values and protein oxidation of leg meat over 8 days period.

Storage Time (T)	Day 0		Day 8		*p*-Values			SE ^1^
Genotype (G)	Hybrid	Polverara	Hybrid	Polverara	(G)	(T)	(G) × (T)	
N.	30	30	30	30				
TBARS values (mg MDA/kg meat)	0.08 ^D^	0.21 ^B,C^	0.14 ^C,D^	0.40 ^A^	<0.0001	<0.0001	0.0330	0.03
Protein oxidation (nmol/mg protein)	1.91 ^B^	3.05 ^A^	2.04 ^B^	3.18 ^A^	<0.0001	0.3812	0.9950	0.14

^1^ Standard error, ^A,B,C,D^ means in the same row with different superscripts significantly differ (*p* < 0.0001).
